# Transcranial Direct Current Stimulation Ameliorates Cognitive Impairment via Modulating Oxidative Stress, Inflammation, and Autophagy in a Rat Model of Vascular Dementia

**DOI:** 10.3389/fnins.2020.00028

**Published:** 2020-01-29

**Authors:** Tao Guo, Jia Fang, Zhong Y. Tong, Shasha He, Yingying Luo

**Affiliations:** ^1^Department of Emergency, The Second Xiangya Hospital, Central South University, Changsha, China; ^2^Department of Neurology, The Second Xiangya Hospital, Central South University, Changsha, China; ^3^Department of Pathology, The Second Xiangya Hospital, Central South University, Changsha, China; ^4^Department of Oncology, The Second Xiangya Hospital, Central South University, Changsha, China

**Keywords:** vascular dementia, tDCS, oxidative stress, inflammation, autophagy

## Abstract

To investigate the potential applications and the molecular mechanisms of transcranial direct current stimulation (tDCS) on cognitive impairment in a vascular dementia (VD) animal model. Sprague-Dawley rats were used in this study. VD rat model was induced by modified permanent bilateral common carotid artery occlusion (2-VO) approach. Anodal tDCS was applied to the animals. Morris water maze was used to analyze spatial memory and navigation ability. The pathological changes in the hippocampal CA1 region and cerebral cortex were examined via Hematoxylin-Eosin staining. The rats were sacrificed for the measurement of the level of superoxide (SOD), glutathione (GSH), reactive oxidative species (ROS), malondialdehyd (MDA), Interleukin (IL)-1β, IL-6, and tumor necrosis factor (TNF)-α level in the hippocampus. Western blot was carried out to measure the hippocampal expression of microtubule-associated protein 1 light chain 3 (LC-3) and p62. Rats with VD have decreased number of neurons in the hippocampus and cerebral cortex, as well as worse cognitive impairment. The proliferation of activated microglia and astroglia, accompanied with attenuation of myelination were observed in the white matter about 1 month after 2-VO operation. These abnormalities were significantly ameliorated by tDCS treatment. Further study revealed that anodal tDCS could suppress the MDA and ROS level, while enhance the SOD and GSH level to reduce the oxidative stress. Anodal tDCS could inhibit hypoperfusion-induced IL-1β, IL-6, and TNF-α expression to attenuate inflammatory response in hippocampus. Moreover, anodal tDCS treatment could alleviate autophagy level. The study has demonstrated a possible therapeutic role of tDCS in the treatment of cognitive impairment in VD.

## Introduction

Vascular dementia (VD) is the second most common cause of dementia after Alzheimer’s disease, accounting for around 15% of cases (O’Brien and Thomas, 2015). Rates of VD rise with age. There have been no effective approved pharmacological treatments available for VD up till now. Chronic cerebral hypoperfusion played a causative role in VD (Du et al., 2017). Previous studies demonstrated that cerebral hypoperfusion could lead to oxidative stress, neuroinflammation, neurotransmitter system dysfunction, mitochondrial dysfunction, disturbance of lipid metabolism, and alterations of growth factor (Du et al., 2017). Oxidative stress plays an important role in cognitive deficits induced by the chronic cerebral hypoperfusion (Chunjiea et al., 2016). Neuroinflammation characterized by Interleukin (IL)-1β, IL-6, and tumor necrosis factor (TNF)-α plays an important role in VD (Belkhelfa et al., 2018). Autophagy, a lysosome-mediated catabolic pathway, contributes to the maintenance of cellular homeostasis (Moloudizargari et al., 2017). Decreasing autophagic activity may contribute to cognitive improvement in rats with VD (Liu et al., 2018; Venkat et al., 2018).

Transcranial direct current stimulation (tDCS) is a non-invasive neuromodulation technique that has been used to modulate brain function (Paulus, 2011). Anodal tDCS could induce long-lasting alterations of cortical excitability and enhance cerebral plasticity, both in experimental animals and humans (Datta et al., 2010). Anodal tDCS could affect synaptic plasticity, modulate the level of oxidative stress, neuroinflammation, and autophagy (Scelzo et al., 2011; Laste et al., 2012; Lu et al., 2015; Lee et al., 2018). Indeed, anodal tDCS is a promising approach for brain diseases associated with impaired neuroplasticity, which is simple to be used and is beneficial for brain function (Podda et al., 2016). It’s speculated that anodal tDCS might exert similar roles in alleviate cognitive impairment in VD. Anodal tDCS has been proven to be beneficial for cognitive function in patients with VD (André et al., 2016). However, the studies of tDCS on VD animal model are limited.

In the current study, we have tried to investigate the possible roles and mechanisms of tDCS on improvement of cognitive impairment of VD rat model. The study could provide some evidences for using anodal tDCS as a potential non-pharmacological treatment for VD.

## Materials and Methods

### Animal Model

**FIGURE 1:**
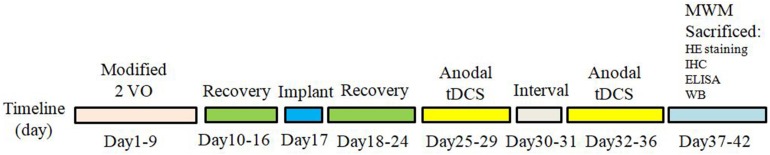
The time schedule of protocols in the present study.

Animal use protocols were approved by the Institutional Animal Care and Use Committee of Central South University in compliance with National Institutes of Health guidelines. Sixty male Sprague-Dawley rats, weighting 250–280 g (6–8 weeks old), were provided by department of laboratory animals in Central South University. The rats were maintained under controlled temperature and humidity (22 ± 3°C and 50%, respectively) with a 12 h light–dark cycle. All efforts were made to limit the number of rats used and to minimize animal suffering. All food and water were provided *ad libitum* throughout the trial. To generate a rat model of VD, permanent bilateral common carotid artery occlusion (2-VO) approach was applied as previously reported (Zhu et al., 2011). Briefly, rats were anesthetized with chloral hydrate; a neck ventral midline incision was made. The common carotid arteries were exposed and then gently separated from the vagus nerve. Carotids were occluded with a 1-week interval between interventions, the right common carotid being the first to be processed and the left one being occluded 1 week later (Cechetti et al., 2010; Mirzapour et al., 2015). The sham operated rats underwent the same procedures without carotid artery ligation. After surgery, rats were left to recover for a period of 1 week. Sixty SD rats were randomly divided into three groups: (1) Sham group: Sham operation group treated with sham stimulation, (2) VD group: VD rat models treated with sham stimulation, (3) tDCS group: VD rat models treated with anodal tDCS. Figure 1 summarized the temporal evolution of the study protocols. Rats that exhibited abnormal behavioral effects during the study, such as seizures, were excluded to avoid any potential impact on the final results.

### tDCS Treatment

One week after surgery, rats were placed in a stereotactic frame after anethetized with chloral hydrate (350 mg/kg). A sagittal incision was made in the scalp. A scalp and the underlying tissues were removed, then the skull was dried with cotton swabs. A custom-made polycarbonate tubes with the inner diameter of 1 mm and the contact area on the skull of 3.14 mm^2^ were stereotactically placed on the sagittal suture with the center of the electrode resting on 2.5 mm posterior to bregma, then the tubes were subsequently attached to the bone surface with a thin layer of non-toxic dental cement (super-bond C&B, Sun Medical, Japan) and a second layer of two-component luting resin (Ketac Cem Plus, 3MESPE AG, Germany) (Pikhovych et al., 2016; Yu et al., 2019). To ensure current flow during stimulation, the hollow implant was kept free of cement. After surgery, the rats were transferred back to their home cages and were allowed to recover for 1 week before undergoing tDCS. After at least 1 week of post-surgical recovery, rats were randomized into two groups receiving anodal tDCS or sham stimulation, respectively. All procedures of tDCS and sham stimulation had identical duration of current fade-in (10 s), fade-out (10 s) and current strength (200 μA), except the stimulation duration (tDCS 30 min, sham 10 s) (Yang et al., 2019; Yu et al., 2019). Anodal tDCS was repeated daily for 5 consecutive days, followed by a 2-day pause, then subjected to another set of 5 stimulation days, resulting in a total of 10 days of tDCS stimulation (Rueger et al., 2012; Pikhovych et al., 2016; Yang et al., 2019). The stimulation was conducted by the same researcher at the same time every day. The anodal electrode was inserted into the polycarbonate tube, which was filled with saturated saline. To avoid debris accumulating in the polycarbonate tube, a cotton ball was placed to seal the tube when not in use (Pikhovych et al., 2016). The cathode electrode was a conventional rubber-plate electrode wrapped by a wet cotton sheath (11 cm^2^) applied over the ventral thorax of the rat by an elastic bandage (Podda et al., 2016). Both anodal and cathodal electrodes were connected to a direct current stimulator (Ruihaikanglian, Jiangsu, China) for electric current stimulation.

### Morris Water Maze Task

The cognitive function was analyzed by the Morris water maze (MWM) test 24 h after the last tDCS stimulation according to previously described protocols (Guo et al., 2017; Schoenfeld et al., 2017). Briefly, a circular water tank with 150 cm diameter and 50 cm depth was filled with 25°C water to a depth of 21 cm. A circular platform (diameter: 10 cm; height: 20 cm) was located in the center of the target quadrant. The navigation trials were conducted for 5 consecutive days. The time taken for the rats to find the platform was recorded as the escape latency. When the rats reached the platform within 90 s, they remained on the platform for 20 s. If the rats failed to find the platform within 90 s, they were gently guided to the platform and left on it for 20 s. The escape latency was recorded as 90 s in such cases. One day after the navigation trial, the platform was removed for a probe trial. The time spent in the target quandrant and the numbers of swimming across the platform site for up to 90 s were recorded using a computer-based image analyzer automatically.

### Tissue Preparation

The brain tissues from rats were collected after completion of the tDCS treatment. All rats were deeply anesthetized using 10% chloral hydrate (400 mg/kg). A subset of rats were perfused transcardially with 0.9% NaCl followed by 4% paraformaldehyde in 0.1M phosphate-buffered saline (PBS). The brains of these rats were removed and post-fixed in the same fixative at 4°C overnight. Then they were immersed consecutively in 20 and 30% sucrose at 4°C until they sank. The remaining post-fixed brains from 2.15 to 5.76 mm behind bregma were embedded in paraffin and then cut into 10 μm thick coronal sections. Hematoxylin and eosin (HE) staining were used to observe any histological changes. Fresh brain tissues were quickly taken and then was fixed in 4% paraformaldehyde for immunocytochemistry or stored at −80°C for enzyme-linked immunoabsorbent assay (ELISA) and western blot.

### HE Staining

Hematoxylin and eosin staining was used to evaluate the morphological changes of the hippocampal region CA1 and cerebral cortex. Sections were then sequentially immersed in hematoxylin for 10 min and in eosin for 1 min (Fan et al., 2018). Four random visual fields from each brain slice were analyzed. Morphological changes of neurons in the hippocampus and cerebral cortex were observed under a microscope at 100 and 400× magnification respectively. The number of surviving neurons in the hippocampal CA1 area and cerebral cortex was quantified under a light microscope at 400× magnification through Image-Pro Plus software.

### Immunohistochemistry

The coronal sections were incubated overnight with anti-glial fibrillary acidic protein (GFAP) antibody, anti-ionized calcium binding adaptor molecule 1 (Iba1) antibody, and anti-myelin basic protein (MBP) antibody. After they were washed, the sections were treated with appropriate biotinylated secondary antibodies. To stain cell nuclei, the sections were incubated with 4′-6-diamidino-2-phenylindole (DAPI) in PBST for 30 min. These sections were visualized by the diaminobenzidine tetrahydrochloride (DAB) and H_2_O_2_. The white matter lesions, astroglia and microglia activations were analyzed in corpus callosum and internal capsule. Quantitative analysis of GFAP, Iba1 or MBP-positive cells present in the sections was carried out under 400 × microscopic magnification by Image-pro Plus software respectively. At least three random microscopic fields from each section (three sections per rat) were calculated. The results were presented in the tiled images. All counts were performed in a blinded fashion.

### Measurement of ROS

The intracellular levels of reactive oxidative species (ROS) were assessed by the probe of DCFH-DA. The hippocampus was separated, rinsed twice with PBS, made into cell suspensions, then incubated in trypsin at 37°C for 25 min. Add ice-cold PBS to stop the reaction. Cells were collected and centrifugated. The sediment was incubated with DCFH-DA containing PBS at 37°C for 30 min. The fluorescene-oxidized deprivation of DCFH-DA was minitored with fluorescene microplate and ROS levels were measured (Jiang et al., 2017).

### Enzyme-Linked Immunoabsorbent Assay

The hippocampus was taken out and put into the homogenate tube after being washed by cold normal saline. Appropriate amount of normal saline at 4°C was added into the tube. Homogenized the tissue, centrifugated at 4000 × *g* for 15 min, then collected the supernatant. The oxidative stress markers in hippocampus were measured: superoxide dismutase (SOD), malonic dialdehyde (MDA), and glutathione (GSH) (Jiang et al., 2017; Al-Amin et al., 2018). The protein levels of interleukin (IL)-1β, IL-6 and TNF-α were evaluated by specific ELISA kits following manufacturer’s instructions (Akang et al., 2019). The commercial kits for examining these parameters were purchased from Nanjing Jiancheng Bioengineering Institute (Nanjing, China).

### Western Blot

The expression levels of autophagy markers in the hippocampus were analyzed by Western blot. In brief, hippocampuses were washed with ice-cold PBS and homogenized in ice-cold RIPA lysis buffer. Total protein concentration was quantified by BCA kit. About 20–40 μg protein samples were loaded onto SDS-PAGE gel for electrophoresis separation and were transferred to PVDF membrane. After blocking, the membranes were incubated overnight at 4°C with primary antibodies. The membrane was then developed by ECL substrate, and images were captured by a computerized system. Quantification was performed using Image J software. The following primary antibodies were used: microtubule-associated protein 1 light chain 3 (LC3) and p62. Insoluble p62 cannot be extracted by conventional methods, so soluble p62 was studied in the present study.

### Statistical Analysis

All statistical analysis was performed using SPSS 22.0 software. The graphs were prepared by GraphPad prism (version 6.0, GraphPad Software, Inc.). Multiple comparisons were analyzed by one-way ANOVA with *post hoc* Bonferroni test. All data were presented as mean ± standard deviation (SD). *P* < 0.05 was considered as statistically significant.

## Results

### Effects of tDCS on Hypoperfusion-Induced Cognitive Impairment

**FIGURE 2:**
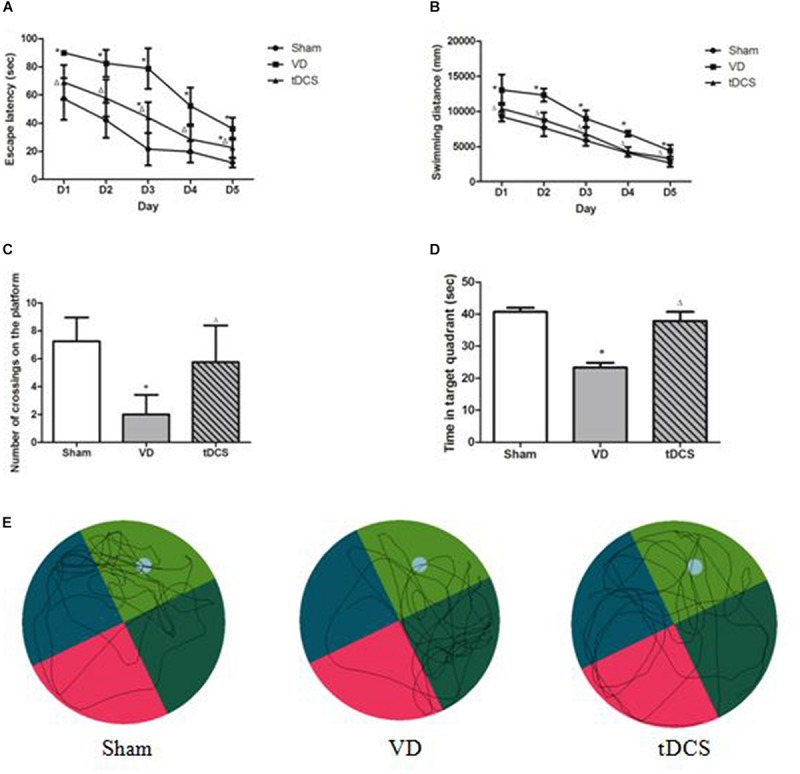
Transcranial direct current stimulation (tDCS) attenuated learning and memory deficits induced by chronic hypoperfusion. **(A)** Escape latency. **(B)** Swimming distance. **(C)** Number of crossings on the platform. **(D)** The time spent in the target quadrant in the spatial probe trials. **(E)** Typical swimming traces of all groups in the spatial probe trials (compared to Sham group, *P < 0.05; compared to VD group, ^△^*P* < 0.05).

In this study, 60 male SD rats were used. Two rats died during the 2-VO surgery. The remaining rats completed all the experiments without any abnormal behavioral effects, such as seizures. All rats successfully finished the task with decreasing latencies to reach the platform. The latencies to find the hidden platform and the swimming distances of the VD rats were significantly longer than those of the rats in the sham group (*P* < 0.05). Furthermore, tDCS induced a significant decrease in escape latencies and swimming distances across 5 days’ MWM training compared with the rats in the VD group (*P* < 0.05) (Figures 2A,B). In the spatial probe trials, rats in the VD model group made significantly fewer crossings of the platform area compared to those in the sham group (*P* < 0.05), whereas rats in the tDCS group crossed the platform area significantly more times than those in the VD group (*P* < 0.05) (Figure 2C). Rats in the VD group spent less time in the target quadrant compared with the rats in the sham group, while tDCS improved the reduction (*P* < 0.05) (Figure 2D). The typical swimming paths recorded in the spatial probe trial of each group were displayed in Figure 2E.

### Effects of tDCS on Neurons in Hippocampus and Cerebral Cortex

**FIGURE 3:**
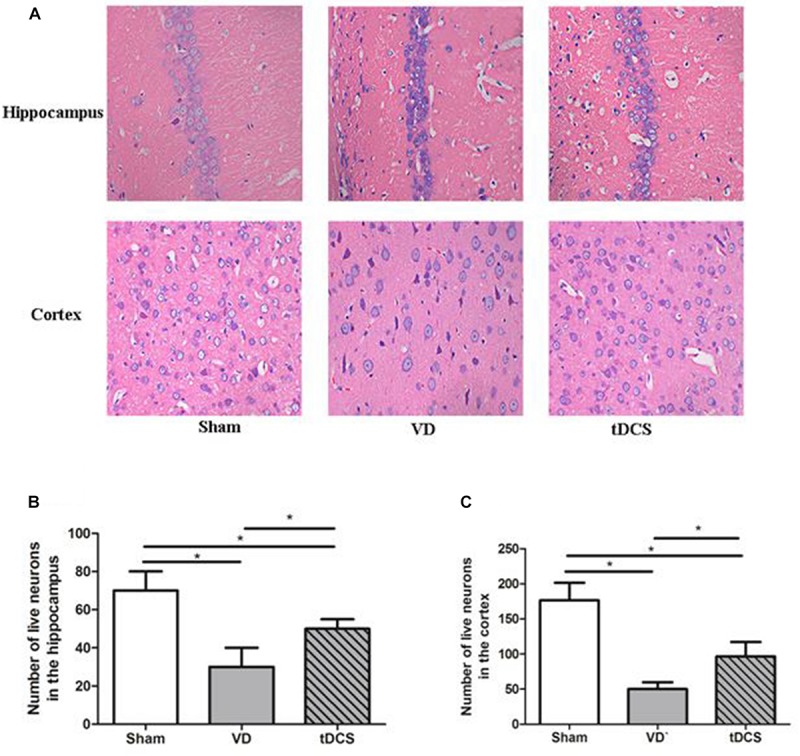
**(A)** Morphological changes in the hippocampal CA1 area and cerebral cortex of rats in each group (HE staining) (×400); Quantification analysis of the numbers of live neurons in the hippocampus **(B)** and the cortex **(C)** of rats from each group (*P < 0.05).

The representative photomicrographs of neurons in hippocampal CA1 region and cerebral cortex were shown in Figure 3. Neurons exhibited regular and compact arrangement in the hippocampal CA1 region and cerebral cortex of sham group rats, with the cytoplasm stained and well-distributed. In the VD group, neuron loss, shrinkage and loose arrangement were observed in the hippocampal CA1 region and cerebral cortex. While in the tDCS group, the neurons demonstrated a nearly normal appearance in the hippocampal CA1 region and cerebral cortex, comparable to those in the sham group. The number of live neurons significantly decreased in the hippocampal CA1 region and cerebral cortex of the VD group than the sham group, while a higher count of live neurons was found in the hippocampal CA1 region and cerebral cortex of tDCS group than the VD group (*P* < 0.05) (Figure 3).

### Effects of tDCS on Demyelination and Glial Activation

**FIGURE 4:**
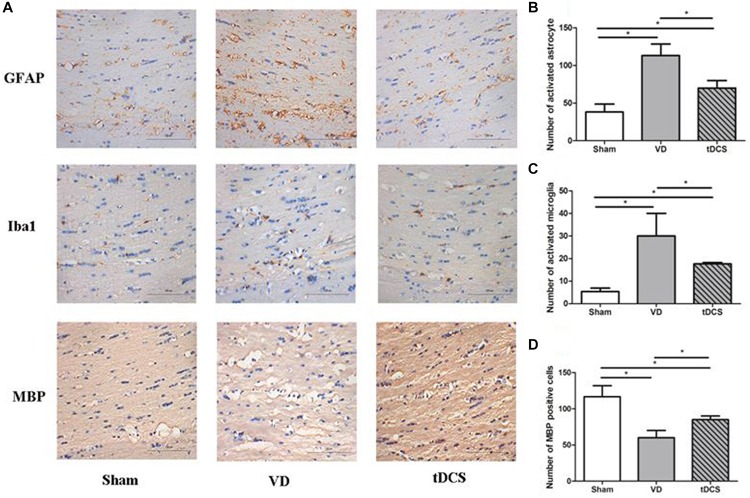
**(A)** Photomicrographs of immunohistochemistry staining for GFAP, Iba1, and MBP in the corpus callosum of rats in each group (×400), scale bar: 100 μm. Quantification analysis of the number of GFAP positive **(B)**, Iba1 positive **(C)**, and MBP **(D)** positive neurons in the corpus callosum of rats from each group (*n* = 6 per group, three sections per rat) (*P < 0.05).

**FIGURE 5:**
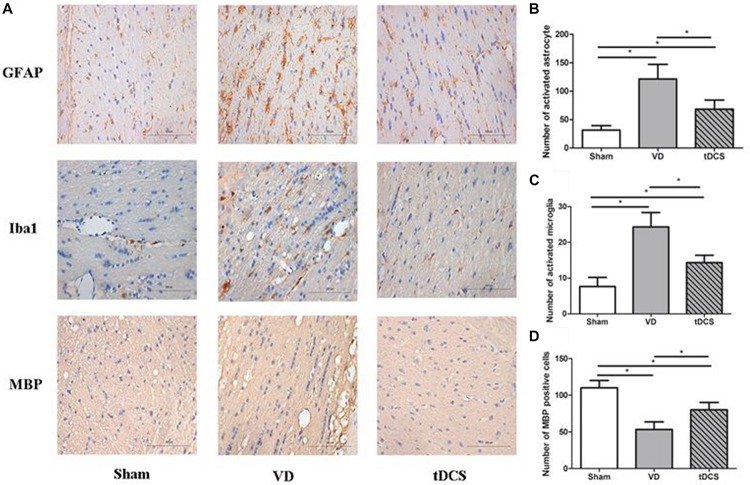
**(A)** Photomicrographs of immunohistochemistry staining for GFAP, Iba1, and MBP in the internal capsule of rats in each group (×400), scale bar: 100 μm. Quantification analysis of the number of GFAP positive **(B)**, Iba1 positive **(C)**, and MBP positive **(D)** neurons in the internal capsule of rats from each group (*n* = 6 per group, three sections per rat) (*P < 0.05).

In the corpus callosum and internal capsule, a significant increase in microglia and astroglia was observed in rats in the VD group compared with rats in the sham group, accompanied by a greater loss of white matter myelin in rats in the VD group. Anodal tDCS attenuated demyelination and glial activation in rats with VD (Figures 4, 5).

### Effects of tDCS on Oxidative Stress Level

**FIGURE 6:**
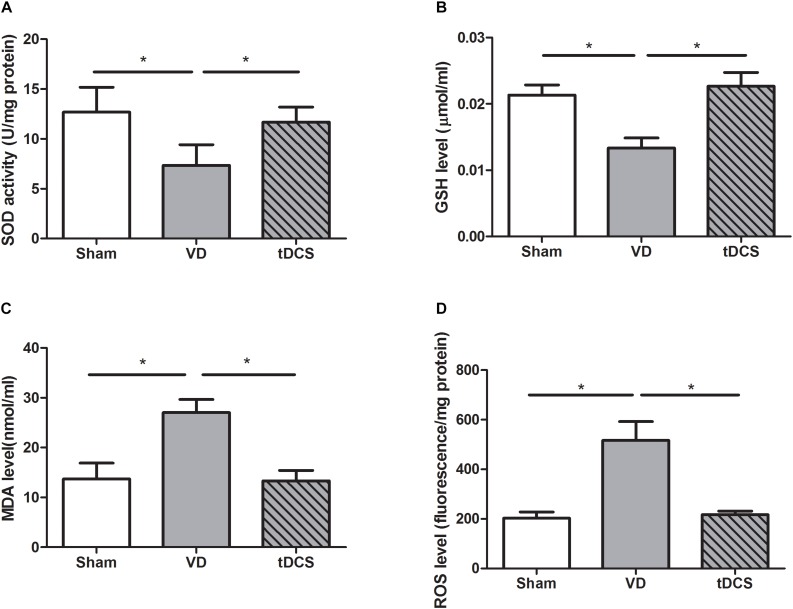
Effects of tDCS on the levels of SOD **(A)**, GSH **(B)**, MDA **(C)**, and ROS **(D)** in the hippocampus of rats from each group (*P < 0.05).

By using commercial kits, we found that SOD (*P* < 0.05) and GSH (*P* < 0.05) reduced significantly, while the levels of ROS (*P* < 0.05) and MDA (*P* < 0.05) increased significantly in the hippocampus of rats with VD. Treatment with tDCS significantly increased the SOD and GSH content. After tDCS treatment, the level of ROS and MDA were suppressed. Thus, tDCS could effectively inhibit the oxidative stress in rats with VD (Figure 6).

### Effects of tDCS on Inflammatory Parameters

**FIGURE 7:**
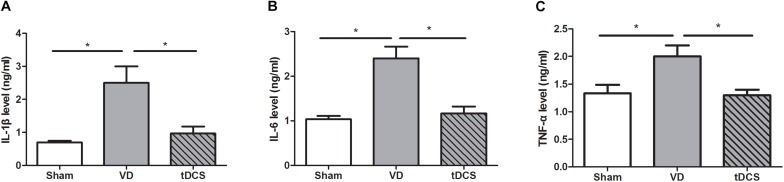
Effects of tDCS on the levels of IL-1β **(A)**, IL-6 **(B)**, and TNF-α **(C)** in the hippocampus of rats from each group (*P < 0.05).

By ELISA quantification, we found that the VD rats had remarkably increased levels of inflammatory factors such as IL-1β, IL-6, and TNF-α (*P* < 0.05). Anodal tDCS significantly alleviated hippocampal protein levels of IL-1β, IL-6, and TNF-α (*P* < 0.05) in the rat model of VD (Figure 7).

### Effects of tDCS on Autophagy Level

**FIGURE 8:**
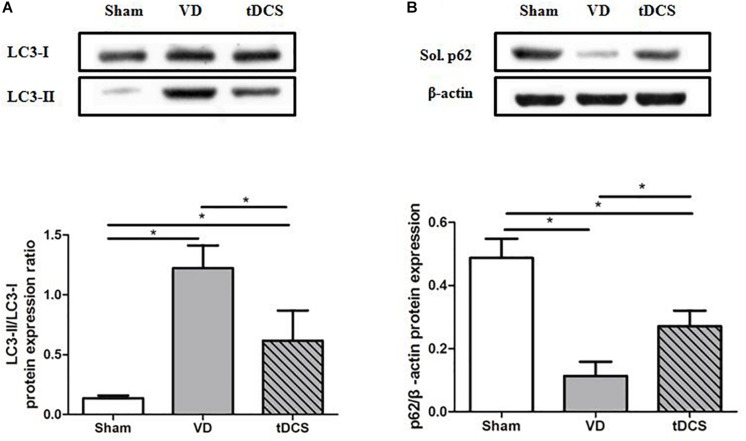
Effects of tDCS on the levels of LC3-II/LC3-I **(A)** and soluble p62 **(B)** in the hippocampus of rats from each group (*P < 0.05).

To determine the effects of tDCS on autophagy, the ratio of LC3-II/LC3-I and the protein expression level of soluble p62 in the hippocampus were analyzed. The ratio of LC3-II/LC3-I was significantly greater in the VD group than that in the sham group (*P* < 0.05). In addition, the expression level of the soluble p62 protein was markedly decreased in the VD group compared with that in the sham group (*P* < 0.05). Anodal tDCS attenuated these changes (Figure 8).

## Discussion

Anodal tDCS has promising potential therapeutic effects for symptoms associated with dementia (Elder and Taylor, 2014). Anodal tDCS exerts positive effects on cognition and brain functions in mild cognitive impairment and major dementias, including Alzheimer’s disease, Pakinson’s disease, however, available studies on VD were limited (Meinzer et al., 2015; Adenzato et al., 2019; Gomes et al., 2019).

Chronic cerebral hypoperfusion is closely related to progressive cognitive impairment in rats (Lin et al., 2011). The most widely used experimental model of VD and chronic cerebral hypoperfusion is 2-VO rats (Du et al., 2017). It’s reported that the modified 2-VO protocol may be more applicable, with similar cognitive impairment and lower mortality rates, compared to the standard 2-VO procedure (Cechetti et al., 2010). The present study established a rat model to reproduce chronic cerebral hypoperfusion by modified 2-VO. Cerebrovascular white matter lesions are caused by chronic cerebral hypoperfusion in VD. The neuropathological changes in VD rat models were characterized by diffuse demyelination and gliosis in the white matter, accompanied with neurodegeneration in the hippocampus and cerebral cortex, which were in accordance with previous reports (Shibata et al., 2004). Hippocampus was sensitive to ischemia, especially the hippocampal CA1 region (Ohtaki et al., 2003).

Brain stimulation techniques can attenuate cognitive impairment in many neuropsychiatric diseases (Chang et al., 2018). The anodal tDCS has been conducted in healthy subjects, Parkinson’s disease, Alzheimer’s disease, multiple sclerosis, depression, and attention disorders, etc. (Lefaucheur, 2016; Chang et al., 2018; Charvet et al., 2018; Manenti et al., 2018). However, studies investigating the effect of tDCS on VD were limited. It’s reported that anodal tDCS of the left dorsolateral prefrontal cortex could improve visual short-term memory in patients with VD (André et al., 2016). The present study demonstrated the beneficial effects of anodal tDCS on the cognitive deficit in a VD rat model. Our study is new in the field of tDCS treatment by demonstrating its anti-autophagy, anti-inflammatory, and anti-oxidant effects in VD.

Autophagy is a self-degradative process that is important for balancing sources of energy. It involves lysosomal-dependent recycling, synthesis, and degradation of intracellular components, thus maintaining the stability of the internal environment. It’s reported that decreasing activity of autophagy may contribute to cognitive improvement in rats with VD (Venkat et al., 2018; Wang et al., 2018). Inhibition of autophagy was beneficial to the hippocampal synaptic plasticity of the VD rat model (Bin et al., 2019). In the present study, the autophagy-lysosomal pathway was activated in the hippocampus of rats with VD. Anodal tDCS could restore the excessive activation of autophagy and help to partly recover the lost learning and memory in VD rats. It’s reported that BDNF expression was enhanced by anodal tDCS, and BDNF could modulate autophagy through the PI3K/Akt/mTOR pathway (Chen et al., 2013). At present, role of autophagy activation in ischemic stroke remain controversial. Autophagy is a double-edged sword. Excessive autophagy can induce autophagic cell death. On the other hand, moderate activation may play a protective role in cell damage.

Increased production of ROS or decreased capacity to clear them could result in oxidative stress. Our data revealed a decrease in the activities of antioxidant enzyme (SOD and GSH) in VD rats, which was attenuated by tDCS. The level of MDA and ROS was reduced by tDCS. In this study, anodal tDCS suppressed oxidative stress induced by chronic cerebral hypoperfusion and protected the hippocampal neurons from further damage induced by overload of ROS. It’s reported that tDCS could reduce oxidative stress in a mouse model of Parkinson’s disease as well (Chengbiao et al., 2015). However, how the altered oxidative status caused by tDCS remained to be determined in the future study.

Neuroinflammation plays an important role in VD. Proinflammatory cytokines IL-1β, IL-6, and TNF-α independently resulted in cognitive impairment (Dugan et al., 2009; Kitazawa et al., 2011; Belarbi et al., 2012). Our data demonstrated that anodal tDCS could significantly restore hippocampus-dependent cognitive deficit induced by neuroinflammation. Previous studies have revealed a decrease in IL-1β and TNF-α after a treatment with tDCS (Leffa et al., 2018; Oliveira et al., 2019). However, how tDCS modulate neuroinflammatory pathways is still not completely understood.

There were some limitations in the present study. First, rats were sacrificed immediately after the last tDCS stimulation. Longer time interval and different time points should be adopted to evaluate the persistence of the effects of tDCS. Second, VD is reported clinically in both males and females. Only male rats were used in the study, therefore a further study is needed to include both male and female rats. What’s more, 2-VO rat model is a VD rat model induced by chronic cerebral hypoperfusion. Rats performed MWM test only about 1 month after 2-VO surgery in the study. Longer time intervals should be adopted to reveal whether the improvements were permanent or not, such as 2 or 3 months after 2-VO surgery.

In summary, our data confirmed that anodal tDCS exerted a neuroprotective effect in the 2-VO rat model. The mechanisms by which anodal tDCS exerted its neuroprotective effects likely involved the modulation of oxidative stress, neuroinflammation, and autophagy. The present study suggested a theoretical basis for the use of anodal tDCS as a potential neuroprotective therapy to improve cognitive impairment of VD.

## Data Availability Statement

All datasets generated for this study are included in the article/supplementary material.

## Ethics Statement

All animal procedures were approved by the Ethics Committee of the Central South University. This study was carried out in strict accordance with the Institutional Animal care and Use Committee of Central South University in compliance with NIH guidelines.

## Author Contributions

JF conceived and designed the experiments. JF, ZT, and SH performed the experiments. JF, TG, ZT, SH, and YL analyzed the data, contributed data, materials, and analysis tools. JF and TG wrote the manuscript.

## Conflict of Interest

The authors declare that the research was conducted in the absence of any commercial or financial relationships that could be construed as a potential conflict of interest.
